# Obesity and Thyroid Cancer Risk: An Update

**DOI:** 10.3390/ijerph19031116

**Published:** 2022-01-20

**Authors:** Fabiana Franchini, Giuseppe Palatucci, Annamaria Colao, Paola Ungaro, Paolo Emidio Macchia, Immacolata Cristina Nettore

**Affiliations:** 1 Department of Clinical Medicine and Surgery, University of Naples Federico II, 80131 Napoli, Italy; fa.franchini01@gmail.com (F.F.); giuseppe.palatucci@unina.it (G.P.); colao@unina.it (A.C.); pmacchia@unina.it (P.E.M.); 2 National Research Council–Institute for Experimental Endocrinology & Oncology ‘Gaetano Salvatore’, 80145 Napoli, Italy; pungaro@ieos.cnr.it

**Keywords:** thyroid carcinoma, obesity, overweight, risk factors, cytokines, insulin, insulin resistance, estrogens, oxidative stress

## Abstract

Thyroid cancer (TC) is the most common endocrine malignancy worldwide and its incidence has increased dramatically in recent years. In parallel, the prevalence of overweight and obesity has also increased, suggesting a possible link between these two diseases. Indeed, low-grade chronic inflammation, altered cytokine levels, insulin resistance, oxidative stress, and hormonal changes that occur in obese patients are all factors that contribute to the occurrence and growth of TC. In this review, the most recent evidence supporting the potential role of the mechanisms linking obesity to TC will be discussed.

## 1. Introduction

Thyroid cancer (TC) is considered the most common endocrine malignancy worldwide. It most commonly affects women and is the second most common cancer in young women. On the tumor incidence scale, TC ranks ninth [1].

The thyroid gland is composed of two distinct endocrine cell types: follicular thyroid cells, which are responsible for iodine uptake and thyroid hormone synthesis, and parafollicular cells, which produce and secrete calcitonin. Thyroid carcinomas can arise from both cell types [2]. According to their histopathological features, TC, which originate from follicular thyroid cells, can be classified into the following categories: (i) differentiated thyroid carcinomas (DTC), which include papillary (PTC) and follicular (FTC) thyroid carcinomas; and (ii) undifferentiated thyroid carcinomas, which include poorly differentiated (PDTC) and anaplastic (ATC) thyroid carcinomas. Medullary thyroid carcinoma (MTC) arises from the neuroendocrine parafollicular C cells of the thyroid [3]. DTC accounts for more than 90% of carcinomas arising from follicular cells and is characterized by an excellent prognosis. PDTC and ATC are rare tumor types, accounting for 5% and 1%, respectively, of TC. They are aggressive tumors with poor prognosis. Finally, MTC accounts for approximately 5% of thyroid carcinomas [3].

In recent years, the incidence of DTC and especially PTC has increased dramatically worldwide [1,4]. Although the exact reason for this is not yet known, improvements in diagnostic techniques have undoubtedly contributed to this increased incidence of DTC [5,6]. Nevertheless, an analysis of data from the Surveillance, Epidemiology, and End Results (SEER) cancer registry from 1980 to 2005 showed an increased incidence of even larger tumors that can be detected without the contribution of advanced diagnostic techniques [7]. Therefore, environmental factors, lifestyle, and comorbidities have been suggested as possible causes for this phenomenon [8].

Many exogenous factors have been associated with the development of TC. The most important are iodine deficiency and radiation, but other factors may also contribute to this disease, including diet, exposure to endocrine-disrupting chemicals (EDCs) or xenobiotics, or other related influences from volcanic areas [8]. These conditions interfere with the epigenetic status of an organism and can affect health status by altering gene expression [9,10]. The best studied risk factor for DTC is radiation exposure, which increases the risk of malignant thyroid disease from 5% to 50% [11]. Other additional risk factors have also been studied, including estrogens [12], cigarette smoking [13], diabetes [14], obesity [15,16], metabolic syndrome [17] and insulin resistance [18]. Many of these risk factors are closely related to an increase in body weight, and therefore it is possible to speculate a role of overweight and obesity in the development and progression of TC. It should be noted, however, that the overall picture is likely to be very muddled. Many of the studies have been limited to examining the effects of a single factor on the development, growth, and expansion of the neoplastic cells, whereas each factor contributing to the disease is also influenced by the complex interplay with other elements. For example, it will be difficult to interpret the significance of the concentrations of a particular molecule without also knowing the concentrations of soluble receptors in serum and the receptor concentrations on the surface of target cells, as well as the relative concentrations of the receptor subtypes of interest. As a result, published studies often provided heterogeneous results, painting a general picture that is complex and only partially addressed.

In recent decades, the prevalence of overweight and obesity has increased dramatically worldwide [19], and the prevalence of DTC expanded in parallel with the increase in excess body weight [20], further supporting the possible correlation between these two conditions [21]. However, in some cases, this correlation seems to be controversial because different methods have been used to assess obesity. According to the World Health Organization (WHO), body mass index or BMI, which is calculated as weight in relation to height (kg/m^2^), is used as a measure of body fat. For optimal health, a BMI between 18.5 and 24.9 kg/m^2^ is recommended. Individuals with a BMI in the range of 25.0–29.9 kg/m^2^ (overweight) are at increased risk for comorbidities, whereas obesity occurs in individuals with a BMI greater than 30 kg/m^2^, who are at moderate to high risk for comorbidities [22]. Indeed, several parameters can be used to assess the clinical relevance of obesity, including visceral or subcutaneous adipose tissue, waist-to-hip ratio, waist circumference, and BMI. The latter is most commonly used but can be inaccurate because it is unable to distinguish adipose tissue from lean mass in some patients. Therefore, none of these parameters alone is suitable for determining the exact status of obesity at the individual level, even when differences in the ethnicity of the individuals studied are taken into account [20].

It has been widely established that elevated BMI is a serious risk factor for a number of chronic diseases, including diabetes mellitus [23], cardiovascular disease [24], chronic kidney disease [25] and musculoskeletal disorders [26]. Obesity is also associated with the development and progression of many cancers [15] and is still considered the second most prevalent, preventable, and modifiable cause of cancer development after smoking [27]. Indeed, the prevalence of cancer is significantly higher in people with obesity [28]. Nevertheless, the possible mechanisms underlying this association are not yet clear, and several obesity-related diseases have been suggested, including hyperinsulinemia and insulin resistance, abnormalities in hormone biosynthesis and hormonal pathways, circadian rhythm disturbances, and alterations in the intestinal microbiome. In addition, obesity has been associated with a chronic and systemic inflammatory state that may be involved in tumor development and progression [29].

In the next pages, we will review the main studies supporting the putative link between obesity and thyroid carcinogenesis and evaluate some of the molecular mechanisms involved in this association.

## 2. Relationship between Obesity and Thyroid Cancer

In recent years, many studies have investigated the relationship between obesity and TC [20,30]. It has been estimated that a five-point increase in BMI and a 0.1-point increase in waist-to-hip ratio increase the risk of TC by 30% and 14%, respectively [31].

Kitahara and coworkers conducted a prospective study based on 22 cohorts participating to the National Cancer Institute Cohort Consortium from North America, Europe, Australia, and Asia. The aim was to examine the association between TC and anthropometric measures (BMI and waist circumference). After a follow-up period of ten years, the authors observed a positive association between the incidence of TC and height, baseline BMI, waist circumference, young-adult BMI and adulthood BMI gain. In addition, baseline BMI and BMI gain were strongly associated with ATC and TC mortality [32].

Kim and coworkers conducted a case-control study in the young Korean population (18 years old) and showed that both men and women with BMI ≥ 25 had a higher risk of developing PTC than those with BMI < 23 [33].

Kitahara and co-authors analyzed a large U.S. database of 457,331 participants aged 50 to 71 years and examined the association between weight status and the incidence rate of PTC. All subjects were asked to complete a self-report questionnaire on health, lifestyle, and BMI. The resulting analysis showed that the risk for PTC was increased in overweight and obese individuals compared with normal-weight participants. Furthermore, the authors demonstrated that obesity was also associated with an increased risk of ATC, suggesting a possible role of obesity in the progression and dedifferentiation of PTC [34].

Finally, a retrospective study conducted on 13,995 Chinese patients with PTC examined the relationships between tissue calcification of PTC, BMI, and tumor invasiveness. The results showed that obesity in PTC patients was associated with a higher risk of tumor invasiveness and tumor tissue calcification [35].

## 3. Molecular Links between Obesity and Thyroid Cancer

**Figure 1:**
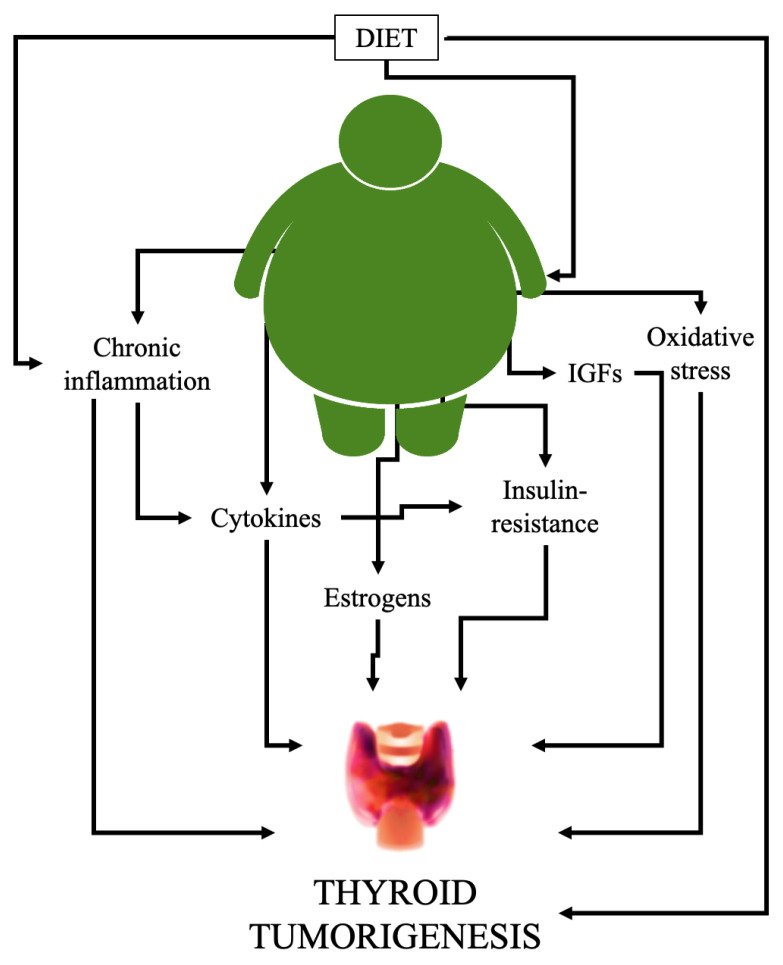
Schematic representation of the main factors linking obesity to thyroid cancer. Diet is the most important factor in the development of obesity, but also plays an important role in influencing TC risk, either directly (i.e.,: iodine deficiency, endocrine disruptors [8]) or through indirect mechanisms (i.e.,: certain pro-inflammatory foods [39]). Obesity promotes additional molecular effects that contribute to induction, growth, and proliferation of TCs. IGFs: insulin-like growth factors.

Recently, the molecular mechanisms underlying the association between the development of TC and obesity have been investigated. In particular, researchers’ interest has focused on the possible role of low-grade chronic inflammation, adipokines [36], deregulation of growth signaling pathways, chronic hyperinsulinemia, estrogens [37,38], altered immune response, and DNA damage from oxidative stress [16] as potential cofactors associated with TC (Figure 1).

Adipose tissue (AT) is a specialized connective tissue formed by fat cells called adipocytes. AT is considered an important regulator of metabolic homeostasis in mammals and is the body’s most effective lipid storage organ. In addition, AT is considered an endocrine organ due to its ability to release various active molecules, called adipokines. These are a variety of bioactive molecules that signal important organs to maintain metabolic homeostasis [40]. Some of the better characterized adipocytokines are adiponectin (APN), leptin, resistin, and many immune system cytokines, such as interleukin-6 (IL-6), tumor necrosis factor-α (TNF-α) and complement factor D or adipsin [36]. Dysregulation of the immune system in adipose tissue of obese individuals leads to chronic low grade inflammation characterized by increased infiltration and activation of innate and adaptive immune cells [41]. In obesity, hypertrophy of adipocyte cells promotes the production of pro-inflammatory factors such as TNF-α, monocyte chemoattractant protein-1 (MCP-1), IL-6, endothelial adhesion molecules, C-reactive protein (CRP) and chemotactic mediators. These are responsible for the infiltration of monocytes and their differentiation into pro-inflammatory M1 macrophages [42]. In turn, M1 macrophages produce and secrete many inflammatory mediators that influence insulin signaling and determine local and systemic pro-inflammatory status [43].

Recently, many studies have addressed the role of pro-inflammatory cytokines produced by adipose tissue and cancer development [36,44]. It has been reported that high concentrations of adipokines promoted by obesity may impair cell proliferation and promote tumorigenesis in the thyroid gland. Indeed, Rehem et al. showed that serum leptin levels were elevated in patients with DTC [45]. In contrast, other studies found lower immunohistochemical expression of adipokines in tumor tissue compared to normal controls [46]. These discrepancies can be partially explained by differences in age, sex, ethnicity, heterogeneity of methodology, and sample size across the different studies.

### 3.1. Adiponectin

One of the most studied adipokines secreted by adipose tissue, is APN. APN is an adipocyte-specific factor, whose primary physiological function is to increase insulin sensitivity. Decreased plasma APN concentrations are associated with insulin resistance, and lower APN concentrations have been observed in various pathological conditions such as obesity, diabetes, and atherosclerosis [47].

APN activates a signaling cascade that leads to various metabolic and immune responses. APN exists in two distinct isoforms that bind to two different types of adiponectin receptors, AdipoR1 and AdipoR2 [48]. The AdipoR1 receptor is expressed almost ubiquitously, whereas AdipoR2 is more abundant in hepatocytes and white adipose tissue.

In recent decades, many studies have investigated the beneficial role of APN on endocrine cancers [49]. APN exerts its effects on tumor tissues influencing multiple intracellular cascades [50,51,52] as well as by modulating tumor angiogenesis [52,53], inflammation [54,55,56] and insulin sensitivity [49]. The role of circulating APN and the association between obesity and TC was recently studied by Zhou and coworkers [52]. APN is drastically decreased in obese individuals, and obesity has been shown to increase the risk of TC in several clinical studies. In 2011, Mitsiades and colleagues showed that circulating APN levels were lower in patients with various forms of TC compared to healthy subjects [57]. A large, multicenter prospective study also confirmed the association between lower APN levels and the presence of TC, especially in female patients [58]. APN levels were also lower in patients with DTC (including PTC and FTC) compared to subjects with benign nodules and controls [59]. Conversely, in a study from Korea, no significant differences in serum APN were found between patients with benign thyroid nodules or PTC [60]. Similarly, decreased APN levels were not associated with tumor size and tumor stages, although the same study highlighted that the prevalence of higher stages of PTC was more common in patients with a BMI ≥ 25 or with metabolic syndrome [61].

Finally, no differences in serum APN levels were observed in patients with MTC compared with healthy controls [62]. An in vitro study conducted by Mitsiades and coworkers showed that thyroid cancer cell lines (SW579 and BHP7) expressed both AdipoR1 and AdipoR2, although the expression levels of the receptors were lower in PTC than in normal thyroid tissue, and the addition of recombinant APN to the culture medium did not significantly alter cell proliferation or survival [57]. These observations were in partial contrast to the data of Cheng and coworkers, who showed that AdipoR1 is weakly expressed and AdipoR2 is moderately expressed in papillary thyroid cancer cell lines K1 and B-CPAP, and that APN activates AMPK phosphorylation in cells after binding to the receptor [63].

In conclusion, as suggested by Zhou and co-authors, it is not possible to demonstrate that circulating APN inversely correlates with increased risk of TC, because the evidence for APN among different BMI stratifications in TC patients is still incomplete [52].

### 3.2. Leptin

Leptin is a pleiotropic hormone produced primarily by adipose tissue and in part by skeletal muscle and the stomach, and plays a critical role in the development of obesity [64]. Together with ghrelin, leptin regulates energy balance and body weight and has a function in energy storage. After its release into the bloodstream, leptin binds to its receptors in the hypothalamus, provides information about the status of the body’s energy stores, and promotes the feeling of satiety [65]. Leptin also affects the expression of cell cycle modulators, cell proliferation, transformation, migration and invasion, stimulation of vascular endothelial growth factor (VEGF), angiogenesis and suppression of anti-inflammatory cytokines [66,67,68]. Its effects are mediated by activation of intracellular signals such as AKT/mTOR/PI3K and ERK /MAPK pathways [69]. Leptin levels correlate with body mass index (BMI) and are higher in individuals with higher BMI and/or greater body fat mass [64]. Leptin resistance and elevated leptin levels are common in obesity [70]. Overexpression of leptin and its receptor (OB-R) has been found in many cancers, including thyroid carcinomas [71,72]. In 2013, Zhang and coworkers evaluated the expression of leptin and its receptors in a cohort of 76 PTC samples and found that they were present in 72.4% (55/76) and 73.9% (56/76) of the tissues examined, respectively [73]. Similar results were also reported by Uddin and colleagues, whose data showed that OB-Rs and leptin were expressed in 80.1% (410/512) and 49.1% (252/513) of samples in a microarray of PTC tissues, respectively [72].

In 2019, Celano and colleagues conducted a translational study to analyze in vitro the molecular mechanisms by which leptin may affect the growth and migration of PTC cell lines. They demonstrated that leptin slightly increased the aggressive phenotype of PTC cells by stimulating proliferation and migration of both TPC and K1 cells [74]. Similar results were obtained by Nigro et al. [75] in both BCPAP and K1 cell lines.

Celano et al. also evaluated the effects of leptin exposure on cells in response to lenvatinib, a protein kinase inhibitor. In these experiments, leptin was not able to affect the effect of lenvatinib [74]. The same study also reported that OB-R transcript and proteins were expressed in all PTC tissues examined, with no significant differences between tumors with the BRAF V600E mutation and BRAF wild-type tumors [74]. Overall, data in the literature suggest that elevated serum and tissue leptin concentrations correlate with thyroid carcinomas [36], and that leptin promotes neovascularization in tumor tissue and induces VEGF expression [76]. However, the current data do not allow to exclude the possibility that elevated leptin levels may also be due to the presence of TC. Therefore, the relationship between leptin and TC is still partially unclear and further studies are needed [36]. In addition, it cannot be excluded that nutritional deficiencies may decrease leptin signaling leading to a lack of satiety, hyperphagia, obesity, inflammation, and in some cases TC.

### 3.3. IL-6 and TNF-α

Increased levels of the cytokines IL-6 and TNF-α are often observed in obese people. These, together with other pro-inflammatory factors, may contribute to promote the development of TC. The role of IL-6 in TC is partially unclear [77]. IL-6 activates the JAK/STAT pathway, which is involved in many metabolic processes, such as energy expenditure, insulin sensitivity, glucose tolerance, adiposity, cell growth and proliferation [78,79,80]. It has been previously reported that the expression of IL-6 is significantly increased in autoimmune thyroid diseases [81] and TC [82]. In addition, patients with benign and malignant thyroid diseases had high levels of IL-6 compared to healthy subjects [83]. This supports the hypothesis that inflammatory processes are related to thyroid diseases [77,80]. An in vitro study has shown that IL-6 promotes the growth and proliferation of anaplastic thyroid cancer stem cells and enhances epithelial-mesenchymal transition (EMT). These effects, mediated by the JAK/STAT3 pathway, contribute to the growth and promotion of metastatic spread of thyroid carcinomas [80].

TNF-α is a proinflammatory factor involved in tumor cytotoxicity and angiogenesis. TNF-α was the first described adipokine produced by adipose tissue, and its circulating levels were elevated in obese and insulin resistant individuals [84]. Kobawala and coworkers found higher levels of TNF-α mRNAs in PTC tissue compared to benign thyroid disease [85]. Later, serum levels of IL-17 and TNF-α were found to be higher in patients with TC and Hashimoto’s disease (HD) compared to healthy controls and to patients with HD without TC [86].

In conclusion, although the data from the literature indicate a strong association between TNF-α and TC [36], the results are still incomplete, and therefore, further studies are needed to define the role of this cytokine in TC [87].

### 3.4. Insulin Resistance and Hyperinsulinemia

Obesity is well known risk factor for diabetes. Obese individuals often have impaired insulin sensitivity, which causes pancreatic beta-cells to promote insulin production, leading to hyperinsulinemia and insulin resistance (IR) [88]. Several studies support the hypothesis of a link between IR and increased risk of many cancers, including TC [89,90,91,92]. Insulin is not only a metabolic hormone but also a cell growth factor that can stimulate the mitogenic cascade by activating the MAPK and mTOR pathways. Its functions are mediated by binding to two receptors: IR-A, which recognizes insulin and more specifically insulin-like growth factors (IGFs) 1 and 2; and IR-B, which is an insulin-specific receptor [93].

The onset of IR can be influenced by several factors, including genetic and epigenetic factors, obesity and diabetes, or exposure to EDCs [94,95]. IR has also been associated with elevated TNF-α levels [84]. All these conditions may increase the risk of TC, and this effect is mediated via the interaction of these elements with several additional risk factors, including iodine deficiency, elevated thyroid-stimulating hormone, estrogen-dependent signaling, chronic autoimmune thyroiditis, and others [92]. In 2009, Rezzonico and colleagues investigated the correlation between IR and DTC [18]. The authors studied a group of twenty women with DTC compared to 20 euthyroid subjects matched for age and BMI. IR was more common in DTC patients with a BMI of >25 compared to control subjects with the same BMI [18]. Similar findings were reported in a recent meta-analysis indicating a higher risk of TC in patients with IR, diabetes, high BMI, and hypertension [91]. Notably, IR was associated with a higher incidence of PTC, while no associations were found between dyslipidemia and TC, although dyslipidemic patients with TC were at higher risk for secondary cancers, including skin melanoma and cancers of the colon, liver, pancreas, ovary, prostate, kidney, urinary bladder, and brain [96].

Further evidence examined the possible relationship between IR and vascularization of thyroid nodules [97]. Wang and coworkers found a positive correlation between HOMA-IR and large nodules, suggesting the hypothesis that IR may stimulate angiogenesis and intra-nodular vascularization, leading to progression of thyroid nodules [97].

Consistent with these findings, it is necessary to expand studies to better understand the relationship between obesity, IR, and neovascularization and, in particular, to define the associations between insulin levels and thyroid carcinomas.

### 3.5. Insulin-like Growth Factors

Insulin-like growth factors (IGFs) play a critical role in normal human physiology and pathological conditions [98]. The IGF system consists of several components: two growth factors (IGF-1 and IGF-2), cell surface receptors (IGF-1R and IGF-2R), six specific high-affinity binding proteins called IGFBP-1-6 and other IGF binding molecules [98,99,100]. The biological effects of IGF-1 are mediated by IGF-1R, a transmembrane protein that possesses a tyrosine kinase domain that, once activated, initiates a cascade of events involving AKT, RAF-1/MEK/ERK proteins, the major signaling pathway involved in cancer proliferation and survival. Deregulation of the IGF axis promotes tumorigenesis in thyrocytes [101].

Many studies have found overexpression of IGF ligands and receptors in various cancers, including breast, lung, pancreatic, colon, prostate, ovarian, and thyroid cancers. This overexpression represents an early event of cell transformation, proliferation, and apoptosis suppressor [102], and is usually associated with poor prognosis [103,104,105,106]. In addition, Schmidt and colleagues showed a positive correlation between the risk of developing DTCs and serum IGF-1 levels [107], and similar results were obtained in a previous study describing the higher expression of IGF-1R in patients with DTCs [108]. Further studies suggest that insulin and/or IGF-1 are also able to enhance the proliferative effect of thyrotropin (TSH), the major physiological promoter of thyroid cell growth. The mitogenic effect of TSH is irrelevant in the absence of growth factors, but is strongly potentiated by IGF-1 [101]. Several lines of evidence suggest that cooperativity and receptor crosstalk also occur in the thyroid and may have functional significance. In vitro studies have shown that IGF-1R upregulates TSH-induced stimulation of thyroid-specific genes, particularly the sodium iodide symporter (NIS), in primary cultures of human thyrocytes. This effect is mediated by a mechanism involving ERK1/2 and/or AKT [109].

The role of IGFBPs has also been investigated for their influence on the development of TC [110,111]. Indeed, in vitro studies have shown that these proteins can have a tumor suppressive effect on IGF activity by sequestering circulating IGF-1 and IGF-2, thereby reducing their binding to IGF-1-2Rs [103,112].

### 3.6. Estrogens

The relationships between gender, obesity, and TC have been largely investigated [16]. Across the world, both obesity and TC are more common in women than men [12,113]. It has been suggested that these gender differences may be influenced by levels of endogenous estrogens, which can act as a growth factor for both benign and malignant thyroid nodules. Leeners and colleagues suggested that estrogens also play an important role in the development of obesity in female [114]. Adipose tissue is involved in the synthesis or conversion of endogenous sex steroids through the action of aromatase [21]. In obese individuals, the increased concentrations and hyperactivation of aromatase lead to an imbalance between estrogens and androgens with increased concentrations of estrogens. This could contribute to thyroid carcinogenesis [16]. Estrogens are steroid hormones that are mainly involved in the regulation of growth, differentiation, and function of reproductive organs. They also have a variety of biological effects in bone and on the cardiovascular and immune systems [115].

Estrogens act through genomic and non-genomic pathways mediated by the nuclear estrogen receptors (ERs), which exist in two different isoforms: ER-α and ER-β. Several studies have demonstrated that in TC cells ER-α is overexpressed, while the expression of ER-β is reduced or absent [116]. In addition, ER-α agonists induce proliferation of TC cells, while increased expression of ER-β or the use of ER-β agonists reduces their growth [117]. The molecular effects of estrogens on thyroid follicular cells are mediated through several pathways, including PI3K/AKT, MEK/ERRK, VEGF and NF-kB [116]. Further, 17-Beta estradiol (E2), the main intracellular estrogen, increases cyclin D1 expression and reduces p27 and beta-catenin expression by binding to ER-α. E2 can also activate the Bcl-2 gene, leading to an increased ability for cell proliferation and survival; and it stimulates ROS production and promotes the hyperexpression of HIF-α [116]. The activation of these pathways confirms that endogenous hormones can influence thyroid development, physiology, and pathological growth of thyroid gland, although the overall effect of estrogen on TC cell growth depends on the balance between ER-α and ER-β expression in the neoplasm. In addition, estrogens determine a variety of genetic and epigenetic changes that modify TC development and invasiveness and stimulate activation of PI3K/AKT and MAPK signaling pathways.

Estrogens also increase angiogenesis in TC by regulating VEGF secretion from thyroid cells [118]. Recently, it has been demonstrated that in PTC of postmenopausal women, the increase in ER-α expression is more pronounced compared to premenopausal women [119]. This finding suggests that ER-α increase may be involved in the aggressiveness of PTC after menopause [116]. However, the expression of ER-α in peripheral blood was significantly increased in women with obesity at both childbearing and perimenopausal ages [120]. Otherwise, a new G protein-coupled receptor, namely GPR30, has been found in some thyroid carcinoma cell lines. This receptor represents an alternative pathway for the transmission of estrogen signaling that can stimulate the growth of cells lacking the classical ERs [121,122].

### 3.7. Oxidative Stress

Obesity is associated with a state of low-grade chronic inflammation characterized by nonspecific activation of the immune system and an increase in inflammatory factors. This condition is responsible for the development of many obesity-related pathological conditions, including hyperglycemia, vascular damage, hyperlipidemia, insulin resistance, and hyperinsulinemia. All of these situations are associated with increased oxidative stress, which contributes to the development and progression of various cancers [123,124].

The term “oxidative stress” is used to indicate a condition characterized by an excess of free radicals and reactive metabolites with harmful effects for the organism [125,126]. Free radicals are molecules that contain one or more unpaired electrons in atomic or molecular orbitals that increase the reactivity of the molecules [127].

Reactive oxygen species (ROS) are derived from oxygen and are generated in cells. ROS are classified into two groups: free radicals, such as superoxide anion (O_2_-) and hydroxyl anion (OH-), and non-radical molecules, such as hydrogen peroxide (H_2_O_2_) [128]. Normally, small or moderate amounts of ROS are produced in cells. They are necessary for many biological functions such as intracellular signaling, modulation of cell death, gene expression, host defense, and hormone synthesis [128,129]. ROS homeostasis is controlled and balanced by the redox regulatory system, which is useful to protect the body from oxidative stress [130]. When the antioxidant system is unable to regulate the ROS balance, the resulting overproduction of ROS is responsible for damage to cells, tissues and organs. This condition is involved in the etiopathogenic mechanisms of many diseases, including TC [126,129].

To date, the role of oxidative stress in TC is unclear, although a link between lower activity of antioxidant systems and excessive production of ROS has been suggested [127]. ROS is responsible for DNA damage through genetic and mutagenic aberrations, which in turn trigger the mechanisms of tumorigenesis through constitutive activation of MAPK and PI3K/AKT signaling pathways. Specifically, several studies suggest that H_2_O_2_ activates the MAPK signaling pathway, which promotes cell growth and proliferation [127]. Other studies have shown that the PI3K/AKT and nuclear factor kB (NF-kB) pathways can be activated by ROS, contributing to the pathogenesis of TC [131]. ROS have also been produced in the synthesis of thyroid hormones [132]. Increased mitochondrial respiration and upregulated ROS production lead to oxidative stress of membrane lipids. In addition, H_2_O_2_ used for thyroid hormone synthesis readily leads to oxidative stress in the thyroid [133]. Oxidative DNA damage is common in the advanced stages of TC, suggesting that oxidative lesions of DNA also contribute to the maintenance of genomic instability in the subsequent stages of tumorigenesis [134]. In 2018, Metere et al. demonstrated lower expression of the selenium antioxidant molecules glutathione peroxidase (GPx1) and thioredoxin reductase (TrxR1) in TC cells compared to normal cells [135]. In addition, decreased catalase expression was found in human thyroid tumors compared to normal thyroid tissue [136,137].

All these findings indicate an imbalanced redox regulatory system and confirm that increased free radical levels are associated with the development of TCs. Finally, micronutrients related to the modulation of ROS production, such as selenium, have been shown to affect apoptosis in follicular thyroid cells [138], and thus, may indirectly influence the survival of TC cells.

### 3.8. Quality of the Diet

Obesity is mainly determined by an imbalance between calories supplied by food and calories consumed. It is, therefore, clear how diet and food choices contribute to the development of obesity. Furthermore, an unhealthy diet plays an important role in the pathophysiology of chronic inflammation [39] and diet has been shown to directly influence inflammatory status [139]. The Dietary Inflammatory Index (DII), originally developed by Cavicchia et al. [140] and updated by Shivappa et al. [141], is a literature review-based score that examines the relationship between various dietary components (foods, nutrients, and flavonoids) and biomarkers of inflammation. A high DII score reflects a more pro-inflammatory diet and has been associated with increased risk of obesity and other chronic diseases, including cancer [39,142]. Recently, two studies examined how elevated DII scores in New Caledonia [143] and in France [144] were associated with a higher risk of DTC, especially when combined with other inflammatory conditions. These new data suggest that diet plays a key role in the etiology of DTC, not only contributing to the global caloric imbalance that leads to overweight and obesity, but also directly modulating the inflammatory process.

Table 1 summarizes the possible mechanisms that modulate the interplay between obesity and TC.

## 4. Conclusions

The prevalence of thyroid carcinoma and in particular the papillary histotype has increased dramatically worldwide in recent years [1,4]. This is partly related to more intensive and sensitive diagnostic procedures [5,6], but also to the influence of various environmental factors, including obesity. Indeed, obesity is considered the second most predictable and modifiable cause of cancer development after smoking, and this condition also plays an important role in the development of TC.

Obesity is associated with a state of low-grade chronic inflammation characterized by nonspecific activation of the immune system, an increase in inflammatory factors and production of various cytokines and adipokines. These elements may directly or indirectly determine cell proliferation and promote tumorigenesis in various tissues, including the thyroid gland. Therefore, a healthy diet with an adequate amount of fruits and vegetables and daily physical activity may be important in reducing the risk of TC. These hypotheses on lifestyle factors deserve attention in future thyroid cancer research.

## Figures and Tables

**Table 1:** Factors that may mediate the obesity effects on thyroid cancers.

Factor	Links to Thyroid Carcinoma	Ref.
Adiponectin	Circulating levels reduced in patients with DTC	[57,58,59]
	Higher expression of APN receptors in thyroid cancer cell lines	[57,63]
Leptin	Presence of leptin receptors in TC	[71,72,73,74]
	Leptin increases the aggressive phenotype of PTC cells,	[74,75]
	Increased circulating levels in patients with DTC	[36]
	Leptin promotes neovascularization and induces expression of VEGF	[76]
IL-6	Increased levels in thyroid carcinomas	[82]
	Increased levels in patients with thyroid diseases	[83]
	IL-6 promotes growth and proliferation of anaplastic thyroid cancer stem cells	[80]
TNF-α	Higher mRNA levels in TC	[85,86]
	Association between circulating levels and TC	[36]
	Possible promotion of growth and metastatic diffusion	[87]
Insulin resistance, hyperinsulinemia	Insulin resistance is more frequent in patients with DT	[18]
	Increased risk of TC in patients with insulin resistance	[91]
	Role in vascularization of nodules	[97]
	Correlation between HOMA index and nodule’s size	[97]
Insulin-like growth factors	Correlation with the risk to develop DTC	[107]
	Higher expression of IGF-1R in patients with DTC	[108]
	IGF binding proteins may have a tumor suppressor effect on DTC	[112]
Estrogens	Overexpression of ER-α in TC	[116]
	Increased angiogenesis in TC through regulating VEGF secretion	[118]
	Increased ER-α expression in post-menopausal TC	[119]
Oxidative stress	Increased ROS production in TC	[127]
	Lower expression of antioxidant molecules in TC cells	[135]
Diet	Higher Diet Inflammatory Index (DII) associated to DTC	[143,144]

## Data Availability

Not applicable.
